# Development and internal validation of a nomogram for predicting grade B/C hemorrhage after pancreaticoduodenectomy

**DOI:** 10.1016/j.clinsp.2026.101134

**Published:** 2026-09-11

**Authors:** Siqing Yi, Jisheng Zhu, Ziyi Ye, Zhichao Li, Zulong Wang, Jiang Wei, Weidong Xiao

**Affiliations:** a Department of General Surgery, The First Affiliated Hospital, Jiangxi Medical College, Nanchang University, Nanchang, Jiangxi, 330006, China; b Institute of Digestive Surgery, Nanchang University, Nanchang, Jiangxi, 330019, China

**Keywords:** Pancreaticoduodenectomy, Postpancreatectomy Hemorrhage, Risk factor, Nomogram, SHapley additive exPlanations

## Abstract

**Objective:**

Postpancreatectomy Hemorrhage (PPH) is a severe complication after Pancreaticoduodenectomy (PD). This study aims to investigate the predictors and develop a nomogram to predict grade B/C PPH.

**Methods:**

Data were collected from patients who underwent PD at the Department of General Surgery of the First Affiliated Hospital of Nanchang University from January 2016 to December 2023. These patients were then randomly divided into a training set and a validation set. Logistic regression analysis was performed to investigate the predictors and develop a nomogram. The model’s discriminative ability was evaluated using the Area Under Curve (AUC) of the receiver operating characteristic, calibration was evaluated using calibration curves, clinical utility was evaluated using decision curve analysis, and generalizability was evaluated by examining model’s performance in the validation set. SHapley Additive exPlanations (SHAP) were used to interpret feature contributions and visualize the model’s decision logic.

**Results:**

Preoperative Prothrombin Time (PT), preoperative Total Bilirubin (TB) (≥171 μmol/L), Postoperative Pancreatic Fistula (POPF), and Postoperative Biliary Fistula (POBF) were independent predictors of grade B/C PPH. The AUC values were 0.848 and 0.721 in the training set and the validation set, respectively. The model demonstrated satisfactory discriminative ability, calibration, clinical utility, and generalizability. SHAP indicated that POPF was the most influential predictor.

**Conclusions:**

Preoperative PT, preoperative TB (≥ 171 μmol/L), POPF, and POBF were identified as independent predictors of grade B/C PPH, and SHAP indicated the importance of POPF. The nomogram based on these predictors can help clinicians predict grade B/C PPH.

## Highlights

• Postpancreatectomy Hemorrhage (PPH) Seriously influences the life safety of patients.
• Pancreatic fistula was the most significant predictor for grade B/C PPH.
• A nomogram was established and internally verified.
• Predictor importance of the nomogram was analysed by SHapley Additive exPlanations.

## Introduction

Pancreaticoduodenectomy (PD) is the standard surgical procedure for tumors of the ampulla of Vater, with a postoperative complication rate ranging from 30% to 50%.^1^ Major complications of PD include hemorrhage, Postoperative Pancreatic Fistula (POPF), Postoperative Biliary Fistula (POBF), and intra-abdominal infection. Among these, Postpancreatectomy Hemorrhage (PPH) represents one of the most critical complications, with an incidence ranging from approximately 3% to 20% and an associated mortality rate of 20% to 50%.^2^ According to the International Study Group of Pancreatic Surgery (ISGPS) criteria, PPH is classified into three grades: A, B, and C. While grade A PPH generally carries a more favorable prognosis, grades B and C are considered life-threatening.^3^ Given the profound challenges in managing PPH, the proactive identification of risk factors and implementation of preventive measures are markedly more effective than post-onset treatment.

A nomogram is a graphical calculating device that provides a visual representation of statistical predictive models. It quantifies the contribution of individual predictor variables and enables the estimation of the probability of a specific clinical outcome. Despite the widespread adoption of nomograms in clinical practice, there is a notable scarcity of nomograms specifically developed for predicting PPH. Although previous studies have developed nomograms for predicting PPH, these models possess certain limitations in their generalizability and clinical applicability. For instance, the model by Birgin et al.^4^ was tailored exclusively to predict POPF-related PPH, and the nomogram established by Li et al.^5^ was specifically designed for PPH following laparoscopic PD. Although the nomogram developed by Duan et al.^6^ using lasso regression demonstrates commendable predictive performance, its incorporation of nine predictor variables may hinder clinical utility in fast-paced or resource-limited settings where a more concise tool is often desirable. Meanwhile, the “black-box” nature inherent to these nomogram models limits their capacity to provide transparent, individualized explanations for specific predictions, thereby hindering clinicians' ability to deeply understand and confidently trust the model's decision-making logic in practice. Moving beyond grade A PPH, which often entails a favorable prognosis, our research targeted the more severe grade B/C PPH after open PD. We developed a nomogram to predict and stratify individual risk of grade B/C PPH. Furthermore, we innovatively incorporated the SHapley Additive exPlanations (SHAP) method to enhance the interpretability of the logistic regression-based prediction model. SHAP provides a unified framework that not only identifies globally important features but also delivers individualized, local explanations for specific predictions, thereby clarifying the decision-making logic behind each instance. Through SHAP analysis, the nomogram is transformed from merely an accurate predictor into an interpretable and trustworthy tool for supporting clinical decision-making.

## Material and methods

### Patients cohorts

This was a retrospective single-center study conducted at the First Affiliated Hospital of Nanchang University, all procedures adhered to the ethical standards of our institution (Project number of the Ethics Committee: IIT20250780) and complied with the principles of the Declaration of Helsinki. This study followed the transparent reporting of a multivariable prediction model for individual prognosis or diagnosis (TRIPOD) statement. All patients had signed an informed consent form. Patients who underwent PD at the Department of General Surgery of our center from January 2016 to December 2023 were included and randomly divided into a training set and a validation set. The training set was further subdivided into two groups based on the presence of grade B/C PPH: the grade B/C PPH group and the non-grade B/C PPH group.

### Inclusion and exclusion criteria

Inclusion criteria: 1) Underwent open PD, including procedures involving reconstruction of the portal vein and/or superior mesenteric vein, or partial resection of adjacent organs, with clear surgical indications; 2) Absence of preoperative dysfunction in vital organs (heart, lungs, brain, or kidneys) and no history of bleeding disorders or coagulopathy; 3) Availability of complete clinical data.

Exclusion criteria: 1) Laparoscopic or robot-assisted PD; 2) Preoperative impairment of vital organs (heart, lungs, brain, or kidneys), or documented bleeding diathesis or coagulation abnormalities; 3) Non-radical resection of the tumor; 4) Emergency surgery; 5) History of previous major abdominal surgery; 6) Incomplete clinical records.

### Observational indicators

Preoperative indicators included: gender, age, history of hypertension/diabetes, Prothrombin Time (PT), serum Total Bilirubin (TB) (with ≥171 μmol/L indicating moderate to severe jaundice),^7^ Aspartate Transaminase (AST), Alanine Transaminase (ALT), albumin level, and preoperative biliary drainage.

Intraoperative indicators included: operation duration, blood loss, transfusion requirement, method of pancreaticojejunostomy, placement of a jejunal nutrition tube, use of external pancreatic drainage, and whether reconstruction of the Portal Vein (PV) or Superior Mesenteric Vein (SMV) was performed, as well as partial resection of adjacent organs such as the liver, colon, spleen, or kidney.

Postoperative indicators included: Postoperative Pancreatic Fistula (POPF) (defined according to ISGPS criteria^8^ as drain fluid amylase level > 3-times the upper limit of normal on or after postoperative day-3, with clinical relevance), Postoperative Biliary Fistula (POBF), intra-abdominal infection, pathological type, lymph node metastasis, postoperative hospital stay.

### Statistical analysis

Normally distributed continuous variables were presented as mean ± standard deviation (x̄ ± s) and compared using the *t*-test. Non-normally distributed continuous variables were presented as median and interquartile range [M (P25, P75)] and compared using the Mann-Whitney*-*test. Categorical variables were presented as frequency and percentage [n (%)] and compared using the Chi-Square test or Fisher’s exact test, as appropriate. Independent predictors of grade B/C PPH were identified through univariate followed by multivariate logistic regression analysis. A nomogram was developed based on the final multivariable model. The discriminative ability of the model was evaluated using the Area Under the Curve (AUC) of the Receiver Operating Characteristic (ROC). Calibration was evaluated using calibration curves generated via the bootstrap method (1000 resamples) and the Hosmer-Lemeshow (HL) test. Clinical utility was evaluated using Decision Curve Analysis (DCA). And the generalizability of the model was verified by evaluating its discriminative ability and calibration in the validation set. SHAP were used to interpret feature contributions and visualize the model’s decision logic. The calculation of SHAP uses the fastshap package in R language, and the visualization uses the shapviz package. All statistical analyses were performed using R-version 4.3.3 and Zstats 1.0 (www.zstats.net), with a two-sided p-value < 0.05 considered statistically significant.

## Results

### General data comparison

A total of 701 patients received PD treatment at the Department of General Surgery of our center from January 2016 to December 2023. According to the inclusion and exclusion criteria, 59 patients were excluded, primarily due to laparoscopic or robot-assisted PD (n = 35), incomplete clinical records (n = 9) and history of previous major abdominal surgery (n = 7). Finally, 642 patients were included in our study. Among enrolled patients, 49 patients (7.63%) developed grade B/C hemorrhage, 7 of whom (14.29%) died as a result. There were 36 cases of intra-abdominal hemorrhage, primarily caused by bleeding from the hepatic artery, gastroduodenal artery, or anastomotic sites. Additionally, 13 patients experienced gastrointestinal hemorrhage, mainly due to ulcers at the gastrointestinal anastomosis. The patients were randomly divided into a training set (n = 450) and a validation set (n = 192) in a 7:3 ratio. The training set was further subdivided into a grade B/C PPH group (n = 33) and a non-grade B/C PPH group (n = 417). Table 1 presents the comparative data between the training and validation sets, as well as between the grade B/C PPH group and the non-grade B/C PPH group. No significant differences in data distribution were observed between the training and validation sets. However, significant differences were identified between the grade B/C PPH group and the non-grade B/C PPH group in terms of preoperative PT, preoperative TB (≥ 171 μmol/L), POPF, POBF, postoperative intra-abdominal infection and postoperative hospital stay (p < 0.05).

**Table 1:** Comparison of clinical data.

Indicators	ALL (n = 642)				Training set (n = 450)		
	Total (n = 642)	Training set (n = 450)	Validation set (n = 192)	p	Non grade B/C PPH group (n = 417)	Grade B/C PPH group (n = 33)	p
Age	59.9 ± 10.1	58.7 ± 10.4	60.3 ± 9.3	0.056	58.7 ± 10.4	58.7 ± 9.9	0.992
Preoperative PT (s)	11.5 ± 3.7	11.3 ± 1.1	11.8 ± 6.6	0.153	11.3 ± 1.1	12.0 ± 1.4	0.006
Preoperative albumin (g/L)	37.4 ± 4.7	37.6 ± 4.7	37.3 ± 4.8	0.567	37.5 ± 4.7	38.3 ± 4.9	0.322
Operation duration (min)	394.2 ± 93.0	393.6 ± 93.7	395.7 ± 91.7	0.791	391.4 ± 92.7	421.2 ± 102.6	0.078
Preoperative AST (U/L)	60.3 (30.2, 125.0)	61.0 (31.2, 129.7)	58.5 (26.0, 118.8)	0.431	62.5 (32.0, 131.0)	57.0 (29.9, 105.0)	0.424
Preoperative ALT (U/L)	54.0 (29.0, 106.0)	55.4 (32.0, 106.8)	48.7 (24.8, 101.4)	0.087	55.0 (32.0, 106.0)	61.0 (34.0, 112.1)	0.660
Blood loss during operation (ml)	400.0 (300.0, 600.0)	400.0 (300.0, 687.5)	400.0 (300.0, 600.0)	0.797	400.0 (300.0, 600.0)	600.0 (300.0, 800.0)	0.209
Postoperative hospital stay (day)	15.0 (12.0, 21.8)	15.0 (12.0, 22.0)	16.0 (12.0, 21.0)	0.703	15.0 (12.0, 21.0)	23.0 (18.0, 34.0)	<0.001
Gender/Male	378 (58.9)	268 (59.6)	110 (57.3)	0.594	251 (60.2)	17 (51.5)	0.328
Pathological type/Malignant	539 (84.0)	378 (84.0)	161 (83.9)	0.963	348 (83.4)	30 (90.9)	0.261
Lymph node metastasis/Positive	172 (26.8)	118 (26.2)	54 (28.1)	0.618	114 (27.3)	4 (12.1)	0.056
Hypertension/Positive	127 (19.8)	88 (19.6)	39 (20.3)	0.826	80 (19.2)	8 (24.2)	0.481
Diabetes/Positive	66 (10.2)	50 (11.1)	16 (8.3)	0.289	47 (11.3)	3 (9.1)	0.924
Preoperative biliary drainage/Positive	192 (29.9)	142 (31.6)	50 (26.0)	0.162	131 (31.4)	11 (33.3)	0.819
Preoperative TB (≥171 μmol/L)	109 (17.0)	74 (16.4)	35 (18.2)	0.581	62 (14.9)	12 (36.4)	0.001
Intraoperative red cell input/Positive	253 (39.4)	174 (38.7)	79 (41.1)	0.556	158 (37.9)	16 (48.5)	0.229
Intraoperative plasma input /Positive	227 (35.4)	153 (34.0)	74 (38.5)	0.270	142 (34.0)	11 (33.3)	0.933
Intraoperative PV or SMV resection and reconstruction or partial resection of liver, colon, spleen and kidney/Positive	50 (7.8)	39 (8.7)	11 (5.7)	0.204	34 (8.1)	5 (15.1)	0.292
Pancreaticojejunosto-my				0.892			0.546
End to End	21 (3.3)	15 (3.3)	6 (3.1)		15 (3.6)	0 (0.0)	
Duct to Mucosa	621 (96.7)	435 (96.7)	186 (96.9)		402 (96.4)	33 (100.0)	
External pancreatic drainage/Yes	69 (10.7)	45 (10.0)	24 (12.5)	0.349	374 (89.7)	31 (93.9)	0.630
Place jejunal nutrition tube/Yes	107 (16.7)	73 (16.2)	34 (17.7)	0.644	71 (17.0)	2 (6.1)	0.100
POPF/Yes	148 (23.0)	98 (21.8)	50 (26.0)	0.240	76 (18.3)	22 (66.7)	<0.001
POBF/Yes	44 (6.9)	35 (7.8)	9 (4.7)	0.156	24 (5.8)	11 (33.3)	<0.001
Postoperative intra-abdominal infection/Yes	59 (9.2)	41 (9.1)	18 (9.4)	0.916	31 (7.4)	10 (30.3)	<0.001
Grade B/C PPH/Yes	49 (7.6)	33 (7.3)	16 (8.3)	0.662	‒	‒	‒

### Logistic regression analysis

Table 2 presents the results of the univariate and multivariate logistic regression analyses comparing the grade B/C PPH group with the non-grade B/C PPH group. Univariate analysis indicated that preoperative PT (OR = 1.69), preoperative TB (≥ 171 μmol/L) (OR = 3.27), POPF (OR = 8.97), POBF (OR = 8.19), and postoperative intra-abdominal infection (OR = 5.41) were significant risk factors for grade B/C PPH. Multivariate analysis identified four independent predictors of grade B/C PPH: preoperative PT (OR = 1.51), preoperative TB (≥ 171 μmol/L) (OR = 3.12), POPF (OR = 6.45), and POBF (OR = 5.48).

**Table 2:** Logitical regression analysis.

Indicators	Univariate analysis		Multivariate analysis	
	OR (95%CI)	p	OR (95%CI)	p
Age	1.00 (0.97 ∼ 1.03)	0.992		
Preoperative PT	1.69 (1.27 ∼ 2.26)	<0.0001	1.51 (1.09 ∼ 2.10)	0.013
Preoperative albumin	1.04 (0.96 ∼ 1.12)	0.322		
Preoperative AST	1.00 (1.00 ∼ 1.00)	0.641		
Preoperative ALT	1.00 (1.00 ∼ 1.00)	0.654		
Operation duration	1.00 (1.00 ∼ 1.01)	0.080		
Blood loss during operation	1.00 (1.00 ∼ 1.00)	0.060		
Gender				
Female	1.00			
Male	0.70 (0.35 ∼ 1.43)	0.330		
Malignant pathology	1.98 (0.59 ∼ 6.68)	0.269		
Lymph node metastasis	0.37 (0.13 ∼ 1.07)	0.065		
Hypertension	1.35 (0.59 ∼ 3.10)	0.482		
Diabetes	0.79 (0.23 ∼ 2.68)	0.702		
Preoperative biliary drainage	1.09 (0.51 ∼ 2.32)	0.819		
Preoperative TB (≥171 μmol/L)	3.27 (1.53 ∼ 6.99)	0.002	3.12 (1.30 ∼ 7.51)	0.011
Intraoperative red cell input	1.54 (0.76 ∼ 3.14)	0.232		
Intraoperative plasma input	0.97 (0.46 ∼ 2.05)	0.933		
Intraoperative PV or SMV resection and reconstruction or partial resection of liver, colon, spleen and kidney	2.01 (0.73 ∼ 5.55)	0.177		
Pancreaticojejunostomy				
End to End	1.00			
Duct to Mucosa	3492,483.93 (0.00 ∼ Inf)	0.988		
External pancreatic drainage	0.56 (0.13 ∼ 2.43)	0.439		
Place jejunal nutrition tube	0.31 (0.07 ∼ 1.34)	0.118		
POPF	8.97 (4.17 ∼ 19.29)	<0.001	6.45 (2.74 ∼ 15.19)	<0.001
POBF	8.19 (3.56 ∼ 18.83)	<0.001	5.48 (2.05 ∼ 14.62)	<0.001
Postoperative intra-abdominal infection	5.41 (2.37 ∼ 12.39)	<0.001	1.04 (0.37 ∼ 2.95)	0.935

### PPH nomogram prediction model

**Fig. 1:**
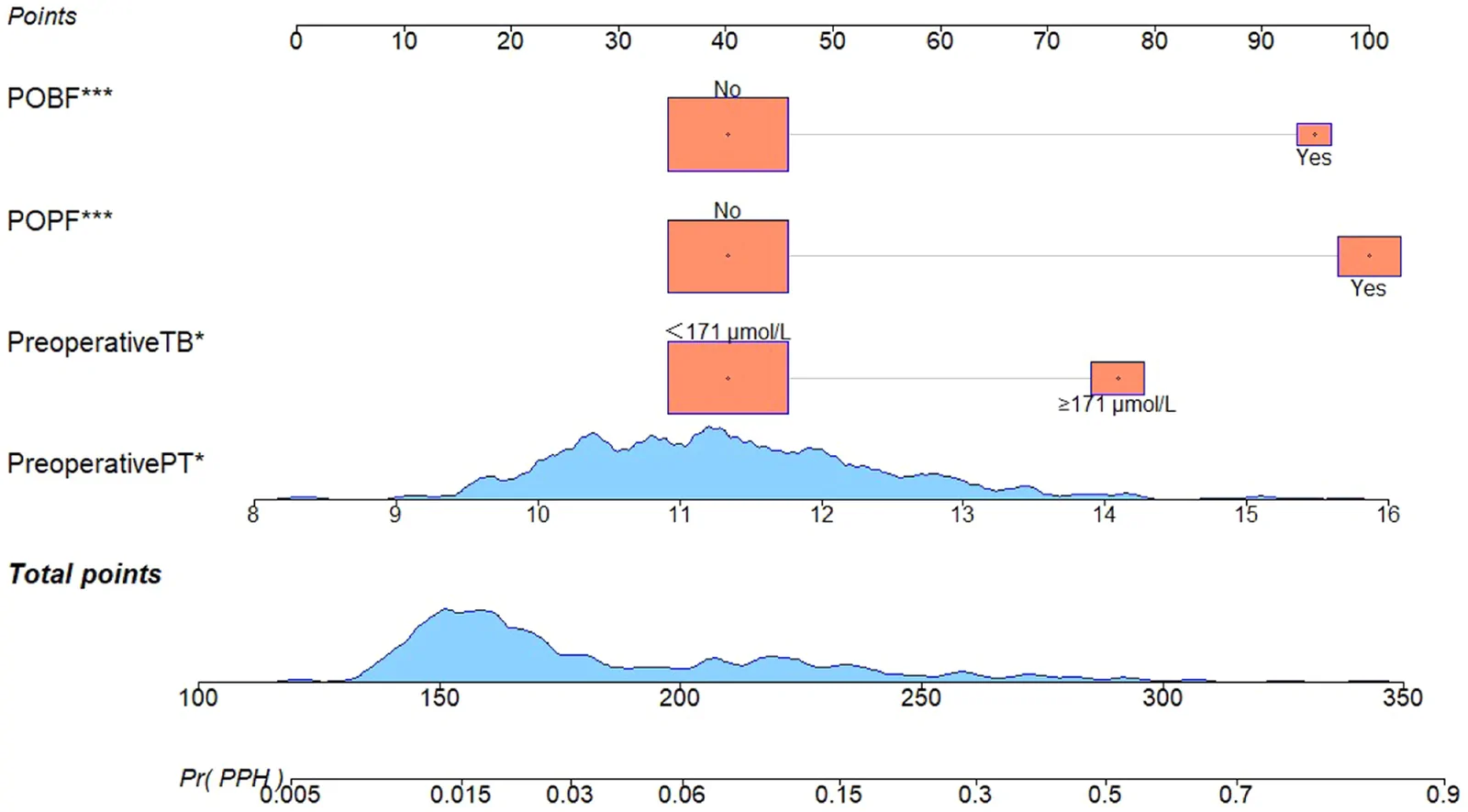
Nomogram prediction model for PPH.

The independent predictors were transformed into a nomogram as shown in Fig. 1. The nomogram demonstrated a specificity of 0.859, a sensitivity of 0.727, and an accuracy of 0.849. The optimal cut-off value of prediction probability for stratifying patients into high and low risk groups was determined to be 0.112.

### Evaluation and validation of the model

**Fig. 2:**
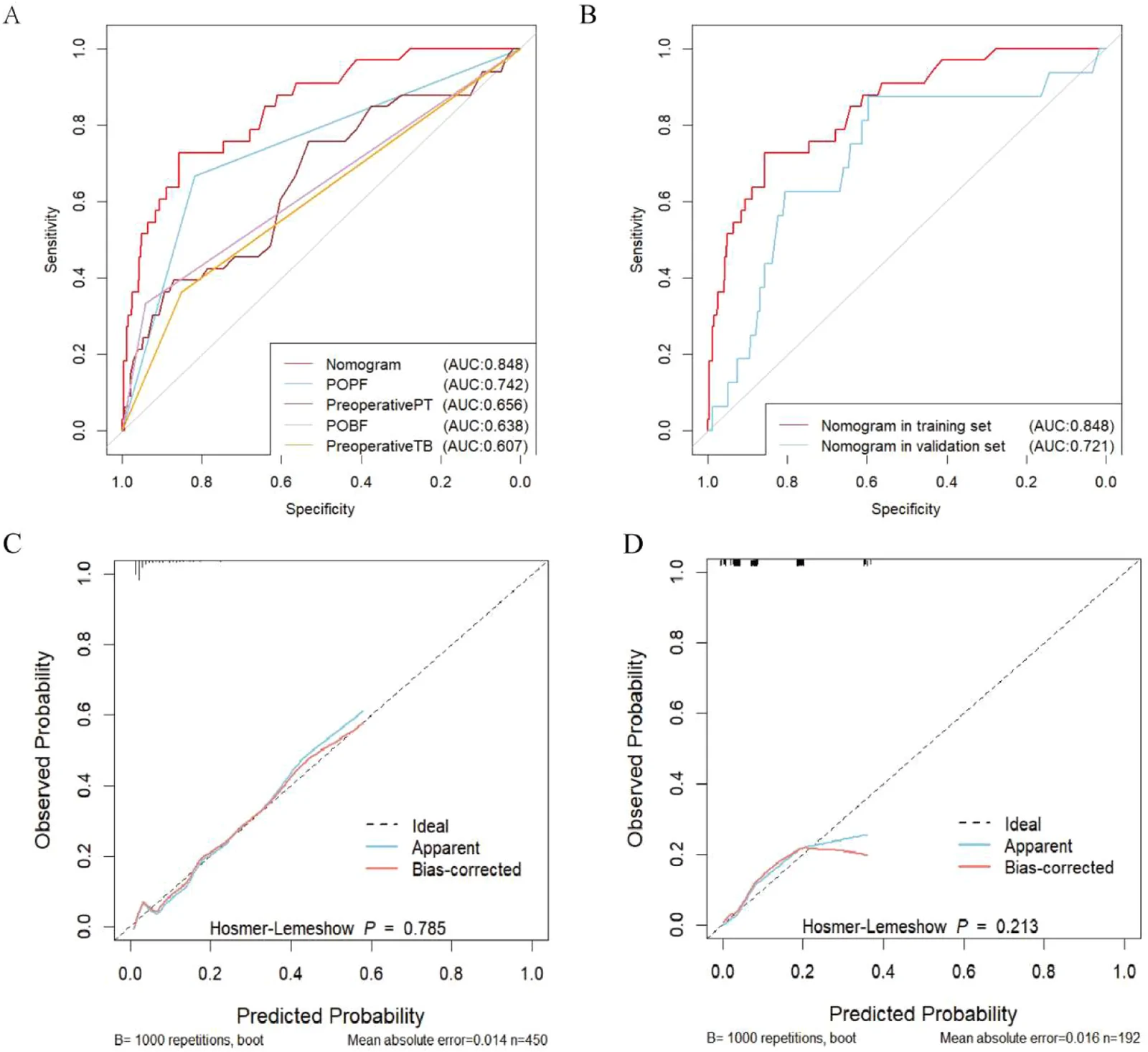
Evaluation and validation of the model. (A) ROC curves of training set. (B) ROC curves of validation set. (C) Calibration curves of training set. (D) Calibration curves of validation set.

The ROC curves of the nomogram are presented in Fig. 2. The nomogram demonstrated higher AUC values in the training set compared to each independent predictor. Specifically, the AUC was 0.848 (95% CI: 0.779∼0.916) for the training set and 0.721 (95% CI: 0.581∼0.861) for the validation set, indicating satisfactory discriminative ability.

Calibration curves are also shown in Fig. 2. The bootstrap-corrected calibration curves closely followed the ideal line in both the training and validation sets. The HL test results were not statistically significant (p = 0.785 and p = 0.213, respectively), with mean absolute errors of 1.4% and 1.6%, supporting good calibration of the model. Furthermore, the performance metrics in the validation set confirm the generalizability of the nomogram.

### DCA curve of the model

**Fig. 3:**
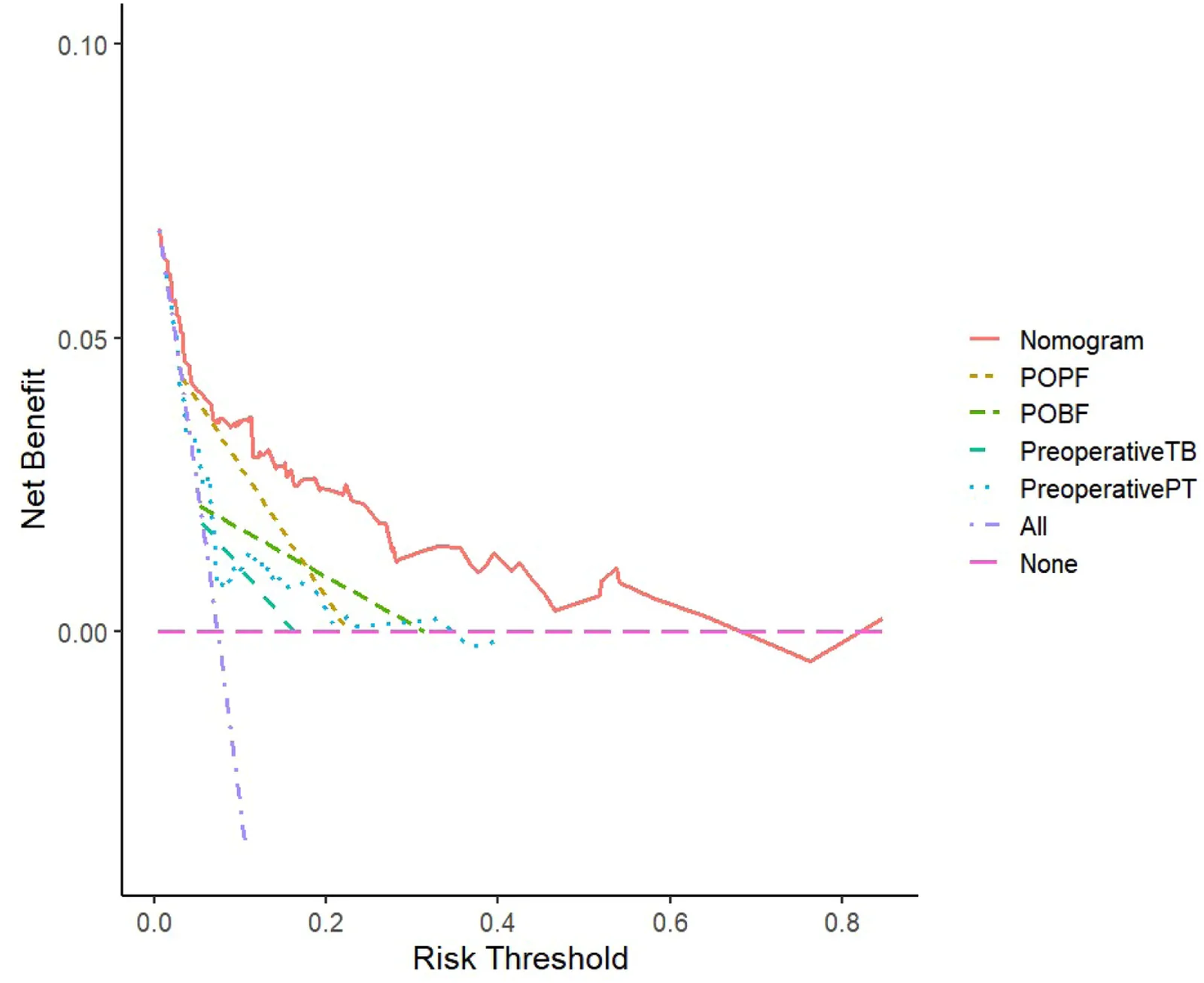
DCA of the prediction model.

The DCA for the nomogram is presented in Fig. 3. The results demonstrate that the nomogram provides a superior net benefit across a clinically relevant range of threshold probabilities.

### SHAP analysis

**Fig. 4:**
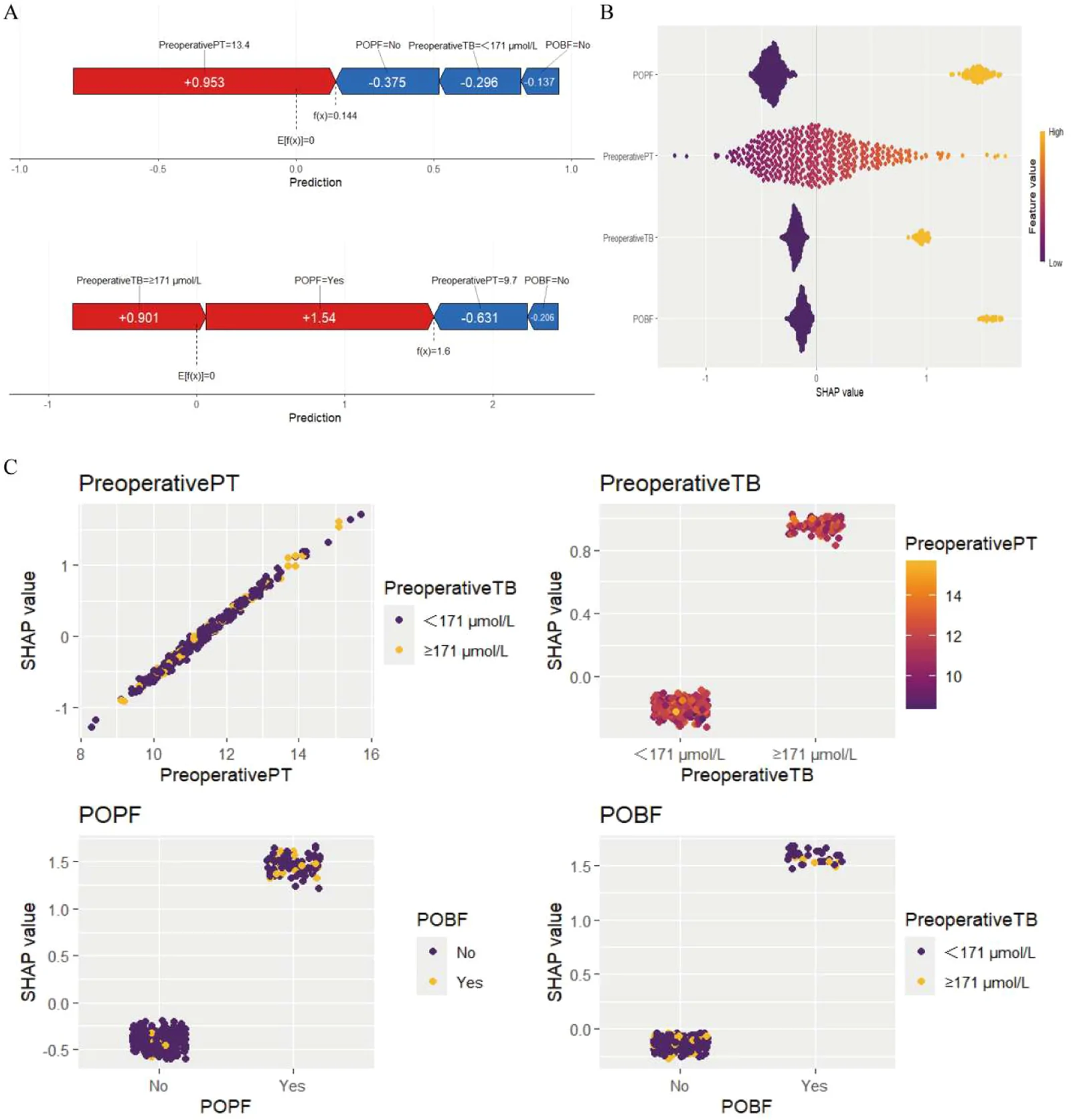
SHAP analysis of the model. (A) Force plots of two random patients. (B) Feature importance plot. (C) Feature dependence plot.

As shown in Fig. 4A, the force plots for two randomly selected patients visually demonstrate the contribution of each predictor to the individual prediction outcomes, illustrating the model's decision-making process. Fig. 4B displays the feature importance plot, indicating that POPF was the most influential predictor. The feature dependency plot in Fig. 4C visualizes the interaction effects between each predictor.

## Discussion

Grade B/C PPH represents a complex clinical condition that poses significant challenges in both diagnosis and management. In this study, 49 cases of grade B/C PPH were identified, with an incidence of 7.63% and a mortality rate of 14.29%, which is consistent with the rates reported in previous literature.^9^^,^^10^ Multivariate analysis identified preoperative PT, preoperative TB (≥ 171 μmol/L), POPF, and POBF as independent risk factors for grade B/C PPH. Patients who developed grade B/C PPH experienced significantly longer postoperative hospital stays compared to those in the control group. Given its notable morbidity and mortality, it is imperative for clinicians to maintain a high level of awareness regarding grade B/C PPH, thoroughly understand its risk factors and underlying mechanisms, and be proficient in its preventive and therapeutic strategies. The development of an accurate predictive model for the early identification of patients at high risk for grade B/C PPH is therefore critically needed to improve patient outcomes.

In patients with obstructive jaundice, bacteria and endotoxins from bile can enter the systemic circulation, potentially leading to severe infections and septicemia. Intestinal bile deficiency, combined with bacterial translocation and increased absorption of enterogenic endotoxins can lead to Systemic Inflammatory Response Syndrome (SIRS) and even Multiple Organ Dysfunction Syndrome (MODS).^11^ Preoperative Biliary Drainage (PBD) serves to alleviate biliary obstruction, restore partial hepatocyte function, and improve coagulation profiles, immune status, and nutritional conditions, thereby enhancing a patient’s tolerance for surgery. However, it is also associated with an elevated risk of postoperative infectious complications.^12^ A cohort study of 202 patients with preoperative TB levels between 40 and 250 μmol/L reported that those receiving PBD had a higher risk of severe complications compared to those undergoing direct surgery, though no significant differences were observed in postoperative hospital stay or mortality.^13^ Additionally, a meta-analysis concluded that while PBD did not affect postoperative hospital stay or mortality, it was associated with an increased overall complication rate, suggesting that routine PBD is not recommended for obstructive jaundice.^14^ Nonetheless, some scholars suggested that PBD may reduce postoperative complications in patients with severe obstructive jaundice.^15^ In our study, no significant association was observed between PBD and grade B/C PPH. Thus, PBD should not be routinely applied in patients with obstructive jaundice, but may be considered on an individualized basis for those with severe cases. As the recovery of liver and immune function following biliary decompression may take 4–8 weeks, a jaundice reduction period of at least 4-weeks is recommended, which could help reduce surgery related complications and shorten postoperative hospital stay.^16^

POPF is the most critical independent risk factor for PPH. Compared to simple pancreatic leaks, POPF following PD is clinically more severe due to the combined leakage of pancreatic juice and intestinal fluid. Beyond the inherent proteolytic activity of pancreatic enzymes, bacterial translocation and spillage of intestinal contents through the fistula tract can provoke life-threatening intra-abdominal infections. This dual pathophysiological mechanism ‒ enzymatic digestion and infective inflammation ‒ synergistically promotes inflammatory edema, exudate accumulation, localized abscess formation, and enzymatic erosion of peripancreatic vessels, ultimately leading to massive hemorrhage.^17^ Subsequent tissue hypoperfusion and hypoxia resulting from hemorrhage can further exacerbate POPF and infection, creating a vicious cycle among POPF, intra-abdominal infection, and hemorrhage. The concurrence of POPF with other complications significantly markedly increases both the incidence of PPH and postoperative mortality.^18^^,^^19^

The development of POPF is primarily associated with a soft pancreatic texture, a small pancreatic duct diameter, and substantial intraoperative blood loss.^20^ During surgery, an appropriate and skilled pancreaticojejunostomy technique should be selected based on individual anatomical conditions, with the quality of the anastomosis being more important than the method employed. In the postoperative period, it is essential to maintain patent abdominal drainage and regularly monitor amylase levels in the drainage fluid for early detection of POPF. Some scholars advocated that wrapping the pancreaticoenteric anastomosis and adjacent vessels with an omental patch can help protect vascular structures and potentially reduce the incidence of PPH.^21^^,^^22^ Somatostatin and its analogues can decrease pancreatic exocrine secretion and reduce the risk of POPF, supporting their routine use in patients with high risk of POPF.^23^ The cornerstone of POPF management is ensuring effective drainage. Image-guided repositioning of abdominal drains may be considered, when necessary, with surgical intervention reserved for cases refractory to nonoperative measures.

Although the prognosis for a simple biliary leak is generally favorable, complex POBF can lead to life-threatening complications such as sepsis and PPH. POBF frequently co-occurs with POPF. Notably, when POBF and POPF coexist, the associated morbidity and mortality of PPH are significantly higher compared to cases involving POBF alone.^24^ The placement of a T-tube in the common bile duct may help reduce the incidence of POBF. The fundamental treatment principle for POBF, similar to that for POPF, is to ensure unobstructed drainage and perform abdominal irrigation if necessary. Biliary drainage by Percutaneous Transhepatic Biliary Drainage (PTBD) can effectively control biliary leakage. In cases where POBF occurs, providing adequate supportive care including close monitoring and correction of electrolyte imbalances resulting from substantial bile loss is crucial.

The management of PPH primarily involves endoscopy, interventional angiography, and reoperation. Early hemorrhage is often attributed to inadequate intraoperative hemostasis, characterized by minimal abdominal adhesions and edema. If conservative measures fail to stabilize the patient, prompt reoperation is recommended to achieve effective hemostasis.^25^ In contrast, delayed hemorrhage typically occurs in a complex postoperative abdominal environment, making reoperation more challenging. Therefore, endoscopic or interventional therapy should be prioritized in such scenarios.^26^ Endoscopic therapy is generally effective for gastrointestinal hemorrhage, while interventional angiography is the preferred approach for intra-abdominal hemorrhage. Sentinel hemorrhage often precedes major bleeding events and usually results from progressive erosion of vessel walls and pseudoaneurysm formation.^27^^,^^28^ Close monitoring upon detection of sentinel bleeding, followed by early diagnostic intervention and timely treatment, can effectively reduce hemorrhage-related mortality.

As an intuitive predictive tool, nomograms demonstrate superior predictive accuracy and flexible evaluation capabilities, leading to their widespread application in prognostic assessments for various malignancies, including hepatocellular carcinoma and breast cancer.^29^^,^^30^ In the field of pancreatic surgery, nomograms have been used to predict outcomes such as POPF,^31^ intra-abdominal infections,^32^ and tumor recurrence.^33^ However, predictive nomograms specifically designed for PPH remain scarce, with existing nomograms primarily focusing on POPF-related PPH or PPH after laparoscopic PD. In this study, we developed a nomogram using our hospital data to predict grade B/C PPH after open PD. It demonstrated a specificity of 0.859, sensitivity of 0.727, and overall accuracy of 0.849. And the nomogram was internally validated and showed satisfactory discriminative ability, calibration, generalizability, and clinical utility. It incorporates only four readily obtainable predictors: preoperative PT, preoperative TB, POPF, and POBF, allowing clinicians to conveniently estimate the probability of PPH.

Moreover, the SHAP method was implemented to elucidate the contribution of each predictive feature and to visualize the model's logic in our study. This provides local interpretability, enabling clinicians to comprehend the rationale behind specific predictions and thus aiding in the formulation of individualized management strategies. Our nomogram includes two postoperative predictors and is mainly used for postoperative dynamic assessment of PPH risk. The best assessment time for the model is 3 to 5days after the operation. This time point represents a convergence for several key clinical decisions, such as whether to initiate oral intake and whether to remove the abdominal drain. It is also the point at which POPF ‒ the most important predictor ‒ can be highly suspected or reliably diagnosed. Then the model can promptly identify individuals with an extremely high risk of PPH, thereby shifting the medical focus from passive response to active prevention. The nomogram’s optimal cutoff value for risk stratification was determined to be 0.112. For low-risk patients (predicted probability < 0.112), clinicians could consider more aggressive treatment strategies to reduce medication and economic burden. High risk patients (predicted probability > 0.112) require close monitoring, entailing strict bed rest, delayed abdominal drain removal, correction of coagulation parameters, close observation of drainage, and advance preparation for potential hemorrhage. It is critical to recognize the dynamic nature of hemorrhage risk, as patients may progress from low to high risk. This underscores the need for reassessment whenever the patient's condition changes. Furthermore, upon detection of sentinel bleeding, preventive gastroscopy or angiographic intervention should be promptly considered.

### Limitations

This study has several limitations. First, as a single-center investigation, it has undergone only internal validation. Therefore, external multi-center validation is necessary before this nomogram can be applied in clinical practice. Second, variations in surgical techniques and experience among operators may have introduced heterogeneity into the included cases. Retrospective design, case screening methods, and the handling of missing data may also lead to potential selection bias. Furthermore, the study focused exclusively on open PD, while laparoscopic and robot-assisted approaches were not considered. To integrate this model into the clinical workflow, prospective studies are still needed for verification. In the future, we plan to conduct multi-center, large-sample prospective studies to develop a predictive model with improved predictive performance and generalizability, ultimately enabling accurate prediction of PPH.

## Conclusions

The independent predictors of grade B/C PPH were preoperative PT, preoperative TB (≥171 μmol/L), POPF, and POBF. The nomogram developed in this study demonstrated satisfactory discriminative ability, calibration, generalizability, and clinical utility, supporting its value as an effective auxiliary tool for predicting grade B/C PPH in clinical practice.

## Data availability

The data sets analyzed during the current study are available upon reasonable request.

## Funding

This research did not receive any specific grant from funding agencies in the public, commercial, or not-for-profit sectors.

## CRediT authorship contribution statement

**Siqing Yi:** Conceptualization, Data curation, Methodology, Visualization, Writing – original draft. **Jisheng Zhu:** Validation, Writing – original draft. **Ziyi Ye:** Investigation, Data curation, Methodology. **Zhichao Li:** Investigation, Data curation, Methodology. **Zulong Wang:** Investigation, Data curation, Methodology. **Jiang Wei:** Data curation. **Weidong Xiao:** Conceptualization, Formal analysis, Supervision, Project administration, Writing – review & editing.

## Declaration of competing interest

The authors declare no conflicts of interest.
